# Hospital-based Surveillance for Pediatric Bacterial Meningitis in the Era of the 13-Valent Pneumococcal Conjugate Vaccine in Ghana

**DOI:** 10.1093/cid/ciz464

**Published:** 2019-09-05

**Authors:** Lorna Awo Renner, Effua Usuf, Nuredin Ibrahim Mohammed, Daniel Ansong, Thomas Dankwah, Jonas Tettey Kusah, Sandra Kwarteng Owusu, Marah Awunyo, Bernard Arhin, Yvonne Addo, John Asamoah, Joseph Nsiari-Muzeyi Biey, Peter Slyvanus Ndow, Archibald Worwui, Madikay Senghore, Bernard Ntsama, Jason M Mwenda, Stanley K Diamenu, Brenda Kwanbana Adams, Martin Antonio

**Affiliations:** 1 University of Ghana School of Medicine and Dentistry, Accra, United Kingdom; 2 Regional Reference Laboratory, Medical Research Council Unit The Gambia at London School of Hygiene and Tropical Diseases, Fajara, United Kingdom; 3 Faculty of Infectious and Tropical Diseases, London School of Hygiene and Tropical Medicine, United Kingdom; 4 Komfo Anokye Teaching Hospital, Kumasi; 5 Korle-Bu Teaching Hospital, Accra, Ghana; 6 World Health Organization (WHO) Inter-country Support Team, Ouagadougou, West Africa, Republic of Congo; 7 WHO Regional Office for Africa, Brazzaville, Republic of Congo; 8 WHO Country Office Ghana, Accra, Ghana; 9 Microbiology and Infection Unit, Warwick Medical School, University of Warwick, Coventry, United Kingdom

**Keywords:** bacterial meningitis, pneumococcal conjugate vaccine

## Abstract

**Background:**

Global surveillance for vaccine preventable invasive bacterial diseases has been set up by the World Health Organization to provide disease burden data to support decisions on introducing pneumococcal conjugate vaccine (PCV). We present data from 2010 to 2016 collected at the 2 sentinel sites in Ghana.

**Methods:**

Data were collected from children <5 years of age presenting at the 2 major teaching hospitals with clinical signs of meningitis. Cerebrospinal fluid specimens were collected and tested first at the sentinel site laboratory with conventional microbiology methods and subsequently with molecular analysis, at the World Health Organization Regional Reference Laboratory housed at the Medical Research Council Unit The Gambia, for identification of *Streptococcus pneumoniae, Haemophilus influenzae,* and *Neisseria meningitidis,* the 3 most common bacteria causing meningitis.

**Results:**

There were 4008 suspected cases of meningitis during the surveillance period, of which 31 (0.8%) were laboratory confirmed. Suspected meningitis cases decreased from 923 in 2010 to 219 in 2016. Of 3817 patients with available outcome data, 226 (5.9%) died. *S. pneumoniae* was the most common bacterial pathogen, accounting for 68.5% of confirmed cases (50 of 73). *H. influenzae* and *N. meningitidis* accounted for 6.8% (5 of 73) and 21.9% (16 of 73), respectively. The proportion of pneumococcal vaccine serotypes causing meningitis decreased from 81.3% (13 of 16) before the introduction of 13-valent PCV (2010–2012) to 40.0% (8 of 20) after its introduction (2013–2016).

**Conclusions:**

Cases of suspected meningitis decreased among children <5 years of age between 2010 and 2016, with declines in the proportion of vaccine-type pneumococcal meningitis after the introduction of 13-valent PCV in Ghana.

## Teaser

The number of children <5 years old with suspected meningitis has decreased after routine 13-valent pneumococcal conjugate vaccine use in the national immunization program in Ghana.

The highest burden of bacterial meningitis are in sub-Saharan Africa [1–4]. The predominant causative pathogens beyond the neonatal period are *Streptococcus pneumoniae*, *Haemophilus influenzae*, and *Neisseria meningitidis*. Besides endemic cases, seasonal epidemics occur during the dry hot season within the meningitis belt in sub-Saharan Africa, caused mainly by *N. meningitidis,* although pneumococcal outbreaks have also been reported [5–8].

Reported case fatality rates (CFRs) for meningitis are high. Global burden estimates for the Africa in 2015 were 15% (uncertainty ranges, 10%–15%) and 28% (uncertainty ranges, 20%–37%) for pneumococcal and *H. influenzae* type b (Hib) CFRs [4], respectively. The highest recorded CFRs were in the African continent [4]. The CFRs for meningococcal meningitis were lower in developing countries, at about 10%–15% [9, 10]. One in 5 meningitis survivors in Africa develop neurological sequelae while in the hospital [11]. Sequelae after discharge from hospital have also been reported [12, 13]. *S. pneumoniae* causes more sequelae than the other 2 bacteria. Globally, the median risk of at least 1 major sequela was estimated at 25%, 10%, and 7% for pneumococcal, Hib, and meningococcal meningitis, respectively [14]. Hearing loss was the most common major sequela, and others include seizures and mental retardation, all of which may be temporary or may result in long-term disability. Bacterial meningitis has huge economic implications for healthcare providers and families [15, 16].

Inappropriate use of antibiotics coupled with antimicrobial resistance make diagnosis and treatment of bacterial infections challenging in developing countries. The World Health Organization (WHO) recommends the inclusion of conjugate Hib vaccines in all infant immunization programs, pneumococcal conjugate vaccines (PCVs), particularly in countries with high childhood mortality rates (ie, >50 deaths per 1000 births in children <5 years old), and large-scale meningococcal vaccination programs in countries with high or intermediate endemic rates of invasive meningococcal disease (>10 or 2–10 cases per 100 000 population per year, respectively) and those with frequent epidemics [17–19]. With support from Gavi, the Vaccine Alliance, these vaccines are now available in many African countries [20].

Ghana introduced Hib-containing (pentavalent) vaccine in 2002 and a 13-valent PCV (PCV13) in May 2012. A MenAfriVac mass vaccination campaign was carried out in 2012 in the 3 northern regions within the meningitis belt. Coverage for 3 doses of Hib has been >80% since introduction of that vaccine and coverage for 3 doses of PCV increased from about 40% in 2012 to >90% in 2016. The national prevalence of human immunodeficiency virus was 2.4% in 2016 [21], The country has a tropical climate with 2 seasons, a wet season from March/April to November and a dry season the rest of the year, with some differences between the northern and southern regions. Malaria is endemic and peaks just after the rainy season. The gross national income per capita for 2016 was $1308 [22].

In 2008, WHO set up the Global Invasive Bacterial Vaccine-preventable Diseases surveillance network to better describe disease epidemiology, measure vaccine impact, and characterize circulating bacterial strains [23]. In Ghana, the surveillance focuses on meningitis at 2 sentinel sites. Here we present 2010–2016 surveillance data from these 2 sites.

## METHODS

### Study Setting

Ghana is located on the west coast of Africa with an estimated population of about 28 million in 2016 [22]. Children <5 years of age represent 20% of the population. The 2 sentinel sites within the Global Invasive Bacterial Vaccine-preventable Diseases network are located at the Korle-Bu and Komfo Anokye teaching hospitals, in the greater Accra region and the Ashanti region, respectively, both outside the meningitis belt. Korle-Bu is the main national referral center and the only public tertiary hospital in Southern Ghana, and Komfo Anokye is the second largest hospital in the country and the only tertiary hospital in the Ashanti region. The former is in the central inland area and the latter on the coast. Both sites are served by the WHO Regional Reference Laboratory (RRL) housed at the Medical Research Council unit The Gambia (MRCG).

### Patients

Data were collected for children <5 years of age presenting at either of the 2 sentinel hospitals with suspected meningitis. Information on demographics, vaccination history (recorded date of vaccine dose), clinical symptoms, antibiotics use before admission, final diagnosis, and outcome at discharge were recorded on a case report form. Cerebrospinal fluid (CSF) specimens were obtained from the children unless clinically contraindicated.

### Laboratory Methods

All CSF specimens were cultured at the sentinel sites for isolation of *S. pneumoniae*, *H. influenzae,* and *N. meningitidis,* using appropriate selective media and following standard methods [24]. Appearance and results of microscopy, gram stain, serology, and culture, when available, were recorded. Isolates from positive samples were sent to the RRL. In addition, aliquots of all negative CSF specimens (ie, CSF with no growth on culture) were also sent to the RRL from 2010 to 2013; from 2014, all CSF specimens were shipped to the RRL, irrespective of the culture result. At the RRL, molecular analysis with quantitative polymerase chain reaction (PCR) was conducted to confirm specific causative pathogen and to serogroup/serotype the species detected [24]. If a bacterial isolate was available, serotyping was conducted using latex agglutination. Whole-genome sequencing was performed on available purified pneumococcal isolates extracted from fresh overnight culture. using methods described elsewhere [25] and displaying the results on a phylogenetic tree with the Web-based Interactive tree of life (iTOL) tool (version 3) [26].

### Case Definitions

A case of suspected meningitis was defined as any child aged 0–59 months admitted with sudden onset of fever (>38.5°C rectal or >38.0°C axillary) and 1 of the following signs: neck stiffness, altered consciousness with no other alternative diagnosis, or other meningeal sign.

Based on laboratory findings, suspected cases were categorized as probable or confirmed. A probable case was one with turbid or cloudy CSF appearance or microscopy and /biochemistry showing a white blood cell count >100/μL or a white blood cell count 10–100/μL and either CSF protein >100 mg/dL or CSF glucose <40 mg/dL. A confirmed case was one with *S. pneumoniae*, *H. influenzae* or *N. meningitidis* isolated from the CSF culture or positive results of serology and/or PCR.

### Statistical Analysis

All data were entered in an Epi Info database tool at the site and forwarded to the RRL, where additional laboratory data were entered. The children’s demographic characteristics and clinical outcomes and the distribution of cases over time were described. Proportions were based on the number of cases with available data for each variable. The variables of age, final diagnosis, and antibiotic use before hospitalization were compared between children who died and those that survived, using χ ^2^ or Fisher exact tests as appropriate. The number of vaccine dose was derived from the number of vaccination dates recorded—1, 2, or 3. The proportions of pathogens causing meningitis were also calculated, and a Poisson regression model was used to assess the effects of the pre-PCV13 (2010–2012) and post-PCV13 (2013–2016) periods on the proportion of PCV13 vaccine-type (VT) isolates.

After CSF processing at the RRL, some results could not be linked to the metadata from the site. In a separate analysis, we included all confirmed pathogens irrespective of the laboratory and demographic data linkage. Statistical analyses were performed using Stata 14 software [27].

### Ethical Review and Approval

Ethical approval was not a requirement in Ghana for routine meningitis surveillance, including drug susceptibility testing of collected isolates as this approved within the routine diagnostic algorithm at the Ministry of Health. However, the surveillance received overarching ethical approval (SCC1188) by the joint MRCG Gambia Government ethics board that allowed the analysis of collected West African isolates at MRCG at the London School of Hygiene and Tropical Diseases.

## RESULTS

Of the 4008 suspected meningitis cases identified from 2010 to 2016 at the 2 sentinel sites, 2561 (63.9%) occurred in children from the Ashanti region, 1160 (28.9%) in children from greater Accra, and 287 (7.2%) in children from other regions. (Table 1). The median (IQR) age of case patients was 12 (2–25) months, and 2154 (53.7%) were male. Among children with available data, 60.4% (915 of 1515) had received antibiotics before admission.

**Table 1.** Characteristics of Children With Suspected Meningitis (N = 4008)

Characteristic	Children, No. (%)
Age, mo	
0–11	1853 (46.2)
12–23	729 (18.2)
24–59	1419 (35.4)
Unknown	7 (0.2)
Sex	
Male	2155 (53.8)
Female	1853 (46.2)
Region	
Ashanti	2561 (63.9)
Greater Accra	1160 (28.9)
Other	287 (7.2)
Case type	
Confirmed	31 (0.8)
Probable	387 (9.7)
Suspected	3590 (89.5)
Final diagnosis^a^	
Pneumonia	83 (2.1)
Meningitis	211 (5.3)
Septicemia	547 (13.6)
Other	512 (12.8)
Unknown	2655 (66.2)
Comorbidity	
No	1138 (28.4)
Yes	157 (3.9)
Unknown	2713 (67.7)
Antibiotics before admission	
No	600 (15.0)
Yes	915 (22.8)
Unknown	2493 (62.2)
Outcome	
Discharged alive	3591 (89.6)
Died	226 (5.6)
Unknown	191 (4.8)
No. of vaccine doses	
PCV13^b^	
1	33 (0.8)
2	49 (1.2)
3	254 (6.3)
Not recorded^c^	3672 (91.6)
Hib vaccine^b^	
1	78 (1.9)
2	141 (3.5)
3	1369 (34.2)
Not recorded^c^	2420 (60.4)

Abbreviations: Hib, *Haemophilus influenzae* type b; PCV13, 13-valent pneumococcal conjugate vaccine.

^a^Final diagnosis according to the clinician.

^b^Doses derived from the actual number of dates recorded for 1, 2, or 3 doses.

^c^No record of vaccination date for all 3 doses.

Ninety-one percent of the children did not have any vaccination date recorded for PCV13, and 60% did not have Hib vaccination date recorded. Among those with records, 8.9% (303 or 3408) and 47.9% (1510 of 3150) received 2 or 3 doses of PCV13 and Hib vaccine, respectively (Table 1). There was no record of MenAfriVac vaccination.

**Figure 1.**
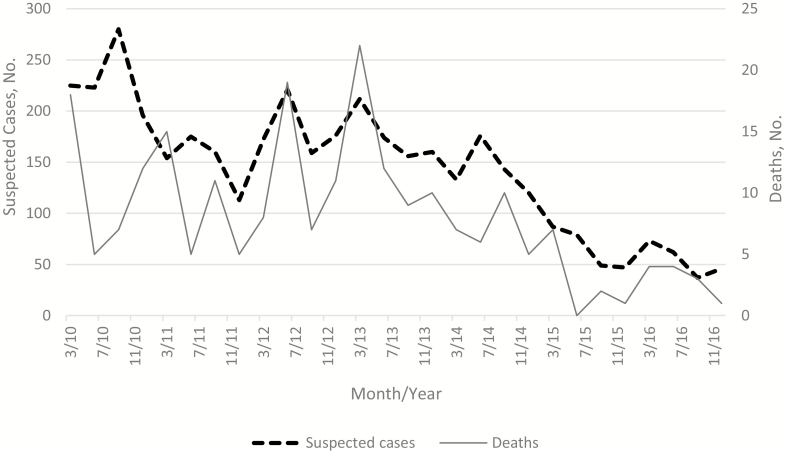
Quarterly distribution of suspected cases and deaths from 2010 through 2016.

The number of suspected cases decreased from 923 in 2010 to 219 in 2016. Monthly trends were observed, with peaks of suspected cases in the first 2 quarters of each year, except for 2010 when the peak was in the third quarter (Figure 1).

Of 3817 children with available outcome data, 226 (5.9%) died (Table 1). The percentage that died did not differ between the pre-PCV13 and post-PCV13 periods (Table 2). The CFR was 4.7% in 2010 and 5.5% in 2016, with the highest number of deaths recorded in 2013 (53 of 226). Among those with a reported final diagnosis from a clinician, septicemia was the most common diagnosis (547 of 1353 [40.4%]), followed by meningitis (211 of 1353 [15.6%]).

**Table 2.** Factors Associated With Death Among Children With Suspected Meningitis

Factor	Suspected Cases			*P* Value
	Patient Survived, No. (%)	Patient Died, No. (%)	Total, No.	
Case type				
Confirmed	25 (86.2)	4 (13.8)	29	.01
Probable	338 (91.4)	32 (8.6)	370	
Suspected	3228 (94.4)	190 (5.6)	3418	
Total	3591 (94.1)	226 (5.9)	3817	
Patient age, mo				
0–11	1653 (93.1)	123 (6.9)	1776	.02
12–23	648 (93.8)	43 (6.2)	691	
24–59	1283 (95.5)	60 (4.5)	1343	
Total	3584 (94.1)	226 (5.9)	3810	
Final diagnosis^a^				
Meningitis	201 (95.3)	10 (4.7)	211	.02
Pneumonia	75 (90.4)	8 (9.6)	83	
Septicemia	529 (96.7)	18 (3.3)	547	
Other	498 (97.3)	14 (2.7)	512	
Total	1303 (96.3)	50 (3.7)	1353	
PCV era				
Pre-PCV era	2045 (94.3)	123 (5.7)	2168	.46
Post-PCV era	1546 (93.7)	103 (6.3)	1649	
Total	3591 (94.1)	226 (5.9)	3817	
Antibiotics before admission				
No	559 (95.6)	26 (4.4)	585	.14
Yes	877 (97.0)	27 (3.0)	904	
Total	1436 (96.4)	53 (3.6)	1489	

Abbreviation: PCV, pneumococcal conjugate vaccine.

^a^Final diagnosis according to the clinician.

**Figure 2.**
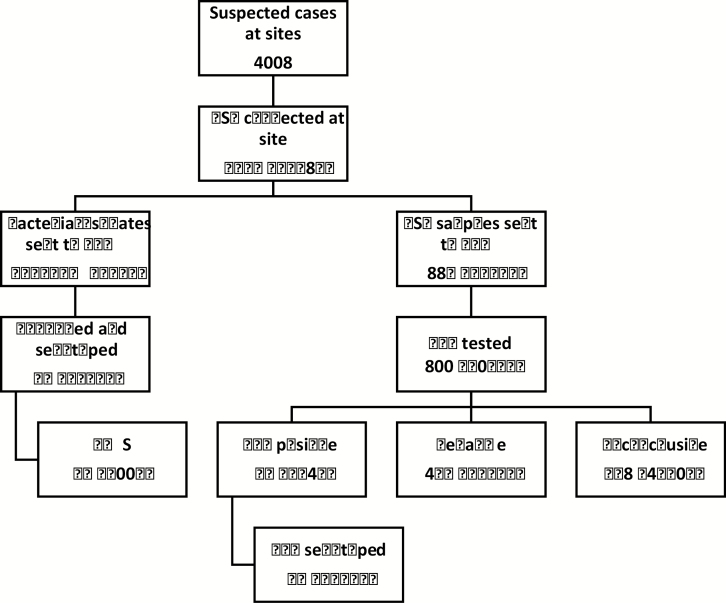
Flow chart of CSF sample processing. One isolate (*Streptococcus pneumoniae*) was not viable at the RRL. Of the CSF samples, 172 could not be linked to metadata from site. CSF cultures were done in 1611 cases, not done in 6, and the status was unknown in 2841. Between 2010 and 2013, culture-positive isolates and culture-negative CSF samples were sent to the RRL, and between 2014 and 2016, all CSF samples were sent to the RRL. Specimens were considered inconclusive if neither a pathogen nor the RNAse P gene was detected. Numbers of confirmed cases are displayed in red. Abbreviations: CSF, cerebrospinal fluid; PCR, polymerase chain reaction; RRL, Regional Reference Laboratory; WGS, whole-genome sequencing.

**Figure 3.**
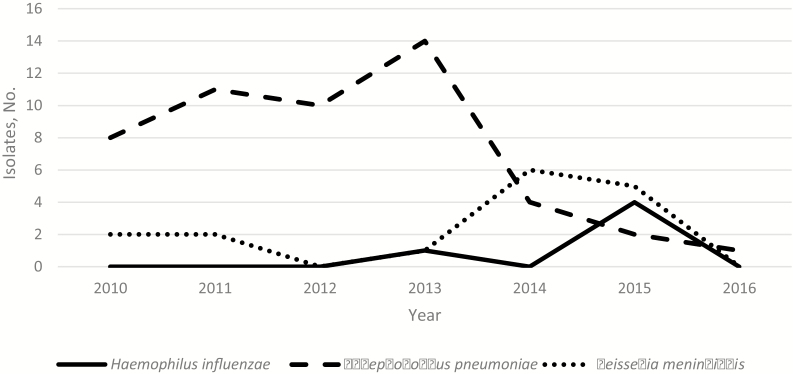
Distribution of confirmed cases by year.

Only 31 cases (0.8%) were confirmed meningitis and 387 (9.7%) were probable meningitis. Twenty-seven bacterial isolates and 887 CSF samples were sent to the RRL (Figure 2). The total number of confirmed cases from the laboratory data was 73, but 42 confirmed cases were not linked to the demographic data. Of the 73 confirmed cases, 50 (68.5%) were *S. pneumoniae*, 5 (6.8%) were *H. influenza*e, 16 (21.9%) were *N. meningitidis,* and 2 were mixed pathogens (1 *H. influenza*e and *S. pneumoniae* and 1 *H. influenza*e and *N. meningitidis*. The annual distributions of confirmed isolates for the 3 bacteria are shown in Figure 3.

Forty-nine confirmed cases were serotyped or serogrouped (Figure 2). Of 5 *H. influenzae* cases typed, 1 was *Hib*, 1 type c, 2 type e and 1 nontypable. Similarly, of the 8 *N. meningitidis* cases typed, 1 was serogroup W, 4 serogroup B, 1 serogroup X, and 1 serogroup Y; 1 isolate was not grouped. Among the 36 pneumococcal meningitis cases, 21 (58.3%) were VT disease. The proportion of VT pneumococcal meningitis cases declined from 81.3% (13 of 16) to 40.0% (8 of 20), with a prevalence ratio of 0.49 (0.20–1.19) (*P* = .12).

**Figure 4.**
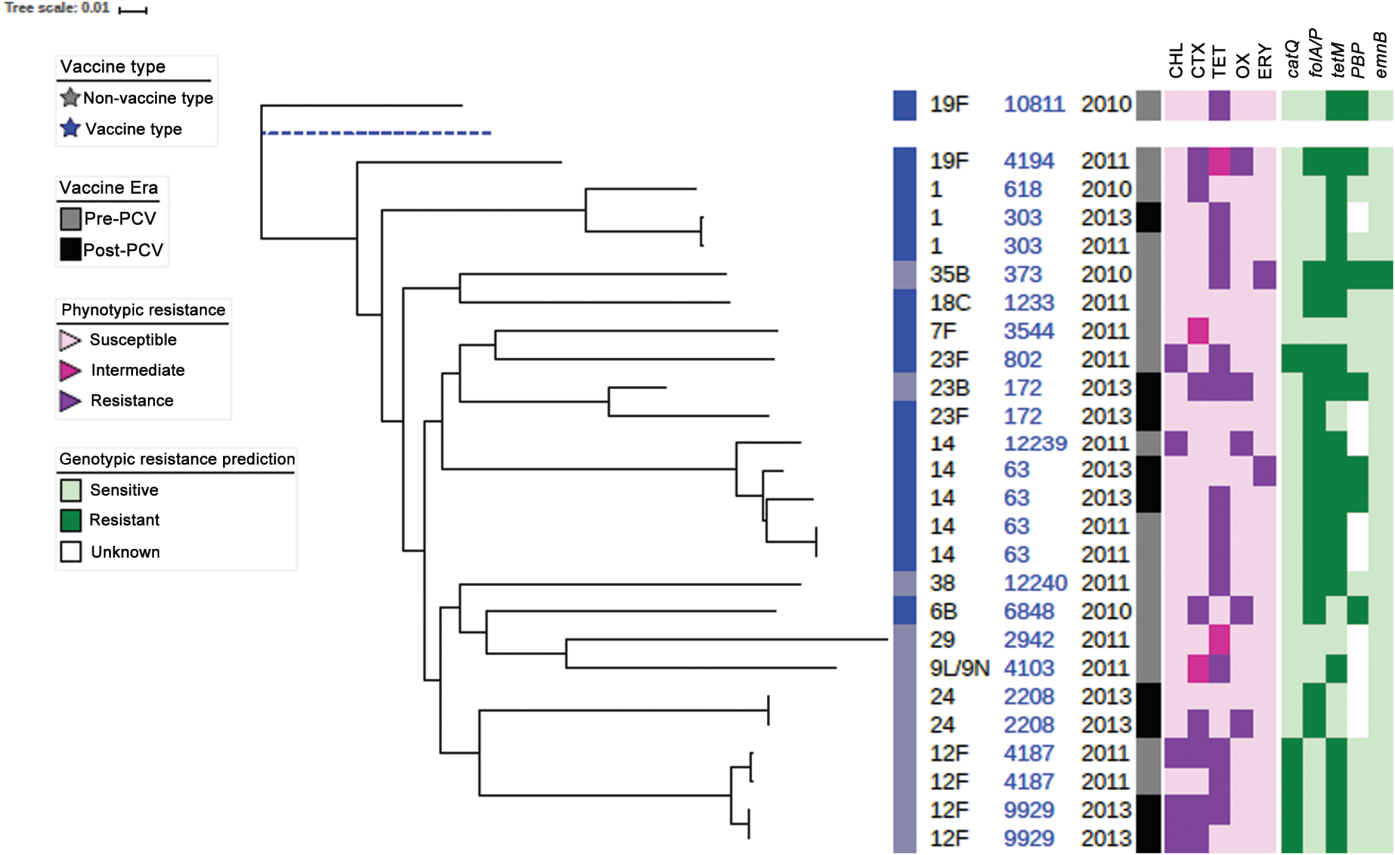
Phylogenetic tree of pneumococcal isolates showing PCV era and antibiogram. Dashed blue line represents the reference genome. Six isolates from blood samples from Ghana included in the tree. Abbreviations: CHL, chloramphenicol; CTX, cotrimoxazole, ERY, erythromycin; OX, oxacillin; PCV, pneumococcal conjugate vaccine; TET, tetracycline.

Whole-genome sequencing of the 26 *S. pneumoniae* isolates that were confirmed at the RRL showed that the predominant lineage was ST 63 (n = 4), from serotype 14 isolates, 2 in the pre-PCV and 2 in the post-PCV era. There was more diversity in the post-PCV era, with 2 ST 9929 from serotype 12F, 2 ST 2208 serotype 24, 2 ST 4103, and 1 belonging to serogroup 9 (Figure 4). An antibiogram showed a cluster of serotype 12F resistant strains (Figure 4).

## DISCUSSION

We report data from hospital-based surveillance in Ghana within the context of conjugate vaccine use for the 3 most common bacteria causing meningitis in sub-Saharan Africa. Between 2010 and 2016, the number of suspected meningitis cases decreased by >70%, whereas the difference in the CFRs was about 1%. *S. pneumoniae* was the leading etiologic pathogen. The proportion of PCV13-type pneumococcal meningitis decreased in the post-PCV era.

Our finding of seasonality of suspected cases of meningitis is consistent with the pattern seen in the meningitis belt with peaks in the first 5 months of the year [5]. From a pooled analysis using country-level data before and after conjugate vaccines, the seasonal timing of bacterial meningitis was between February and March, during the dry season for 19 countries in the African meningitis belt. The study further suggested that the pattern has not changed in the postvaccination era [28].

PCVs have been shown to decrease VT pneumococcal disease in population-based studies from the Gambia, Mozambique and South Africa [29–31]. We observed that the proportion of PCV13-type pneumococcal meningitis decreased by 51% from the pre-PCV (2010–2012) to the post-PCV (2013 and 2016) era. Recently, concerns have been raised regarding the persistence of VT and emergence of non-VT in disease and carriage after 5 years of PCV13 in the United Kingdom [32]. Carriage data in 1 study in the subregion, 5 years after PCV13 has also shown persistence of VT and emergence of non-VT [33]. Because carriage is a precursor for disease, there is a need to continue monitoring, particularly in Africa where the disease burden was very high before vaccine introduction.

After the introduction of the Hib vaccine, Hib disease has diminished immensely [34–36]. Continued surveillance will help determine whether there are selected populations harboring Hib [37]. Regarding nontypable *H. influenzae*, a review in the Hib era reported that there was no convincing evidence of a substantial or sustained increase after vaccination [38].

Although vaccination data were incomplete for many children, it was not surprising that we did not observe any record of MenAfriVac receipt, given that mass vaccination campaigns were conducted in Northern Ghana in 2012 and the national rollout of MenAfriVac started only in 2016. We found 1 case of *N. meningitidis* serogroup W and no *N. meningitidis* serogroup A. The latter has decreased remarkably after the campaigns that started in 2010 in sub-Saharan Africa [5]. In a recent outbreak in Ghana, the main serogroup was W [8].

We have shown here that continued surveillance is useful to understand the epidemiology of bacterial meningitis and to describe changes in the etiologic pathogen as countries introduce new conjugate vaccines. This analysis has some noteworthy limitations. For some variables, the data were incomplete, but the quality of data collection is assured because the surveillance system has been monitored by WHO, with improvements to data management that include training and an external quality assessment program for all laboratories, including the RRL [39, 40]. Although CSF specimens were collected from most children with suspected meningitis, definitive diagnoses were lacking because an etiologic pathogen was rarely isolated. 

Use of antibiotics before hospitalization was common and may have contributed to the low yield of bacterial isolation from CSF specimens. Although one-third of patients had no reported prior antibiotic use, this does not exclude antibiotic consumption. One study in the Gambia showed high urine antibiotic activity among patients who reported no antibiotic use before presentation to the hospital [41]. Moreover, in more than half of the cases (62.5%) it was not known whether the child had received an antibiotic before admission. Not all the CSF specimens were tested with PCR, and limited bacteriological facilities at the hospital laboratories may be a contributing factor for the low CSF yield.

We compared VT disease in 2 periods, before and after vaccine introduction, without a translation phase to account for vaccine uptake. The vaccine was introduced in 2012 without a catch-up campaign, and coverage was about 40% [42]. By including the year of introduction in the prevaccine period, we have diluted the effect of the impact. The numbers were small, and our prevalence ratio was not statistically significant. Our estimates of total vaccination doses irrespective of age are likely to be underestimates, because many children did not have recorded vaccination history data. National coverage for 3 doses of Hib and PCV13 were both reported to be >90% in 2016 [42].

In conclusion, suspected and consequently confirmed cases of meningitis decreased from 2010 to 2016. The number of deaths has also decreased by about 70% over the surveillance period. After the introduction of PCV13 in 2012, we showed a decrease by 50.8% in PCV13-type pneumococcal meningitis, suggesting potential vaccine impact. Only 1 case of Hib meningitis and no *N. meningitidis* serogroup A was observed during the surveillance period, a finding likely to be related to the introduction of Hib in 2002 and the location of the sentinel sites outside the African meningitis belt regions. Continued surveillance is essential to evaluate long-term vaccine impact and to monitor changes in the circulating bacterial strains causing disease.

## Notes

***Invasive Bacterial Disease (IBD) writing group.*** The writing group includes B. K. A., M. S., E. U., A. W., Uzochukwu Egere, Akram Zaman, Catherine Okoi, Florian Gehre, Leopold Tientcheu, N. I. M., Felix Dube, P. S. N., J. M. M., Sambou Suso, Sheikh Jarju, Dam Khan, Chinelo Ebruke, Rowan Bancroft, and M. Antonio.

***Author contributions.*** M. Antonio and J. M. M. established the World Health Organization (WHO) Regional Office for Africa–supported Paediatric Bacterial Meningitis Surveillance Network in West Africa. M. A. supervised the overall network, including setting up the sentinel surveillance system. L. A. R., D. A., T. D., S. K. O., M. Awunyo, and B. A. clinically investigated and recruited the patients at the sentinel sites, collected demographic data and performed microbiological testing at sentinel sites, and shipped cerebrospinal fluid and bacterial isolates to WHO collaborating center (WHOCC) at the Medical Research Council unit The Gambia (MRCG) for confirmatory testing and molecular analysis, supervised by B. K. A. and M. Antonio. Along with the IBD writing group, E. U., B. K. A., and M. Antonio developed the analysis plan and helped analyze and interpret data. L. A. R., E. U., B. K. A. and M. Antonio drafted the manuscript. N. I. M. provided statistical advice. All authors helped interpret the findings and write the final manuscript.

***Acknowledgments.*** The authors thank the WHO Country Office of Ghana and WHO Inter-country Support Team for coordination, advice, and support throughout the surveillance period. They also thank the surveillance participants and their families in Ghana and the staff members and students at the WHOCC MRCG, as well as the IBD writing group for their advice and input.

***Financial support.*** Financial support for sentinel site surveillance was provided by the Ghana Ministry of Health, Gavi - the Vaccine Alliance, through a grant to the WHO for the African Paediatric Bacterial Meningitis Surveillance Network.

***Supplement sponsorship.* This supplement was supported with funds from Gavi, the Vaccine Alliance through The World Health Organization and the CDC Foundation, and The Medical Research Council Unit The Gambia at the London School of Hygiene and Tropical Medicine.**

***Potential conflicts of interest.*** E. U. reports fees related to a GlaxoSmithKline consultancy (2014–2017), outside the submitted work. All other authors report no potential conflicts. All authors have submitted the ICMJE Form for Disclosure of Potential Conflicts of Interest. Conflicts that the editors consider relevant to the content of the manuscript have been disclosed.
